# Supplementary material for: β-arrestin mediates communication between plasma membrane and intracellular GPCRs to regulate signaling
Source: Commun Biol. 2020 Dec 18;3:789. doi: 10.1038/s42003-020-01510-2 (PMC7749148; doi:10.1038/s42003-020-01510-2)
Supplement: Supplementary file 2 — Supplemental Information [file 42003_2020_1510_MOESM2_ESM.pdf]

Supplementary Figures

β-arrestin mediates communication between plasma membrane and intracellular GPCRs to regulate signaling

Maxwell S. DeNies[1], Alan Smrcka[2], Santiago Schnell[1,3,4], Allen P. Liu[1.5,6,7]

[1] Cellular and Molecular Biology Program, University of Michigan, Ann Arbor, Michigan, USA

[2] Department of Pharmacology, University of Michigan Medical School, Ann Arbor, Michigan, USA

[3] Department of Molecular & Integrative Physiology, University of Michigan Medical School, Ann Arbor, Michigan, USA

[4] Department of Computational Medicine & Bioinformatics, University of Michigan Medical School, Ann Arbor, Michigan, USA

[5] Department of Mechanical Engineering, University of Michigan, Ann Arbor, Michigan, USA

[6] Department of Biomedical Engineering, University of Michigan, Ann Arbor, Michigan, USA

[7] Department of Biophysics, University of Michigan, Ann Arbor, Michigan, USA

Corresponding author:

A.P.L: allenliu@umich.edu; 2350 Hayward Street, University of Michigan, Ann Arbor, Michigan 48109. Tel: +1 734-764-7719.

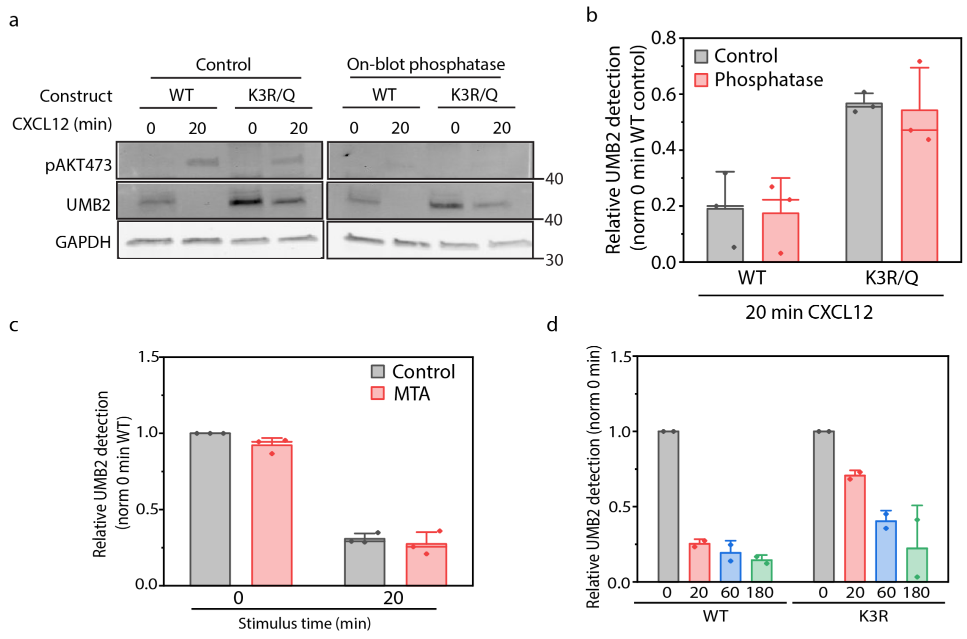

Supplementary Fig. 1: UMB2 antibody characterization. **a** Representative western blot of on-blot phosphatase experiment with WT and K3R/Q mutant CXCR4 constructs. The K3R/Q mutant receptor is unable to be ubiquitinated at the canonical C-terminal CXCR4 ubiquitination sites. The on-blot phosphatase blot was incubated with Lambda Phosphatase to remove phosphate groups from phosphorylated protein residues. Phospho-AKT S473 probing was included as a phosphatase treatment positive control. **b** Quantification of on-blot phosphatase western blotting experiments. Data was normalized by dividing the UMB2 detection at 20 min by the 0 min time point for either the WT or K3R/Q receptor. **c** Flow cytometry analysis of arginine protein methylation activity on CXCR4 UMB2 detection. As illustrated, cells were treated with methylthioadenosine (MTA 200 µM), an arginine protein methyltransferase inhibitor. UMB2 detection was normalized to total CXCR4 fluorescence and secondly to 0 min control detection. **d** Flow cytometry analysis of UMB2 antibody detection for an extended CXCL12-stimulus time course using WT or K3R mutant CXCR4. UMB2 detection was normalized to total CXCR4 fluorescence and secondly to 0 min detection for either WT or K3R mutant receptor. All experiments were conducted in RPE cells overexpressing WT CXCR4 and stimulated with 12.5 nM CXCL12 for the stated time course. Individual data points from each experiment are plotted; mean, SD, median line. Complete raw blots are shown in Supplementary Fig. 11.

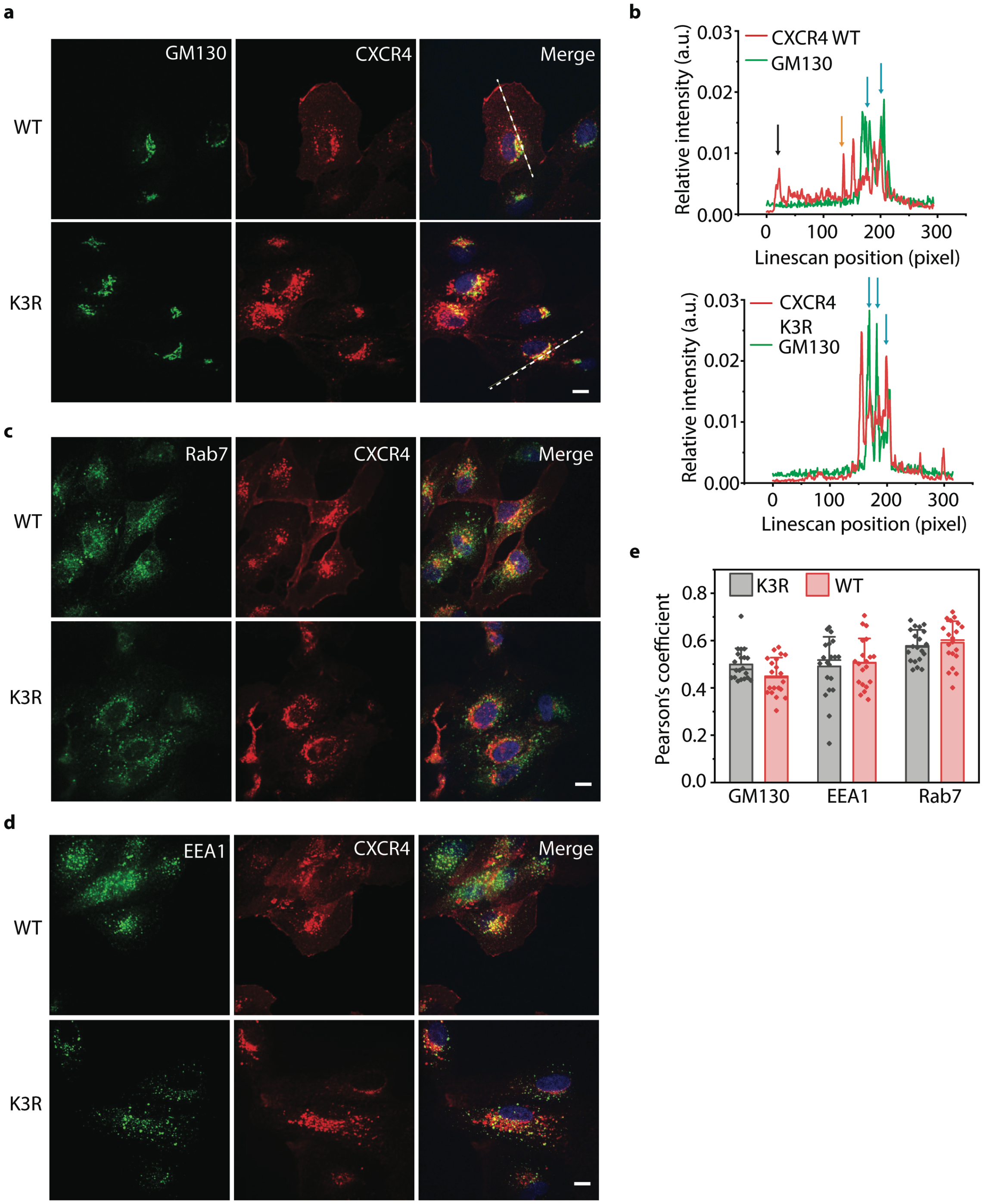

Supplementary Fig. 2: Characterization of CXCR4 intracellular localization. **a** Representative confocal microscopy images of WT and K3R CXCR4 colocalization with GM130. **b** Corresponding line scan analysis of CXCR4 (WT and K3R) and GM130 localization. Data was normalized to total fluorescence in a cell. Blue arrows are indicative of colocalization between CXCR4 and GM130. The black arrow indicates

plasma membrane localized CXCR4 (WT only) and the orange arrow illustrates that CXCR4 puncta also localizes to other intracellular compartments. **c-d** Representative confocal microscope images of WT and K3R CXCR4 colocalization with Rab7 and EEA1. **e** Colocalization analysis of WT and K3R CXCR4 with GM130, Rab7, and EEA1 using Pearson's correlation coefficient. All experiments were conducted in RPE cells overexpressing WT CXCR4 and stimulated with 12.5 nM CXCL12 for the stated time course. Individual data points from each experiment are plotted; mean, SD, median line. All analyses were conducted in ImageJ. Pearson's correlation coefficient was calculated using the JACoP plugin from n = 20 images for each condition and cell line. Scale bar is 10 μm.

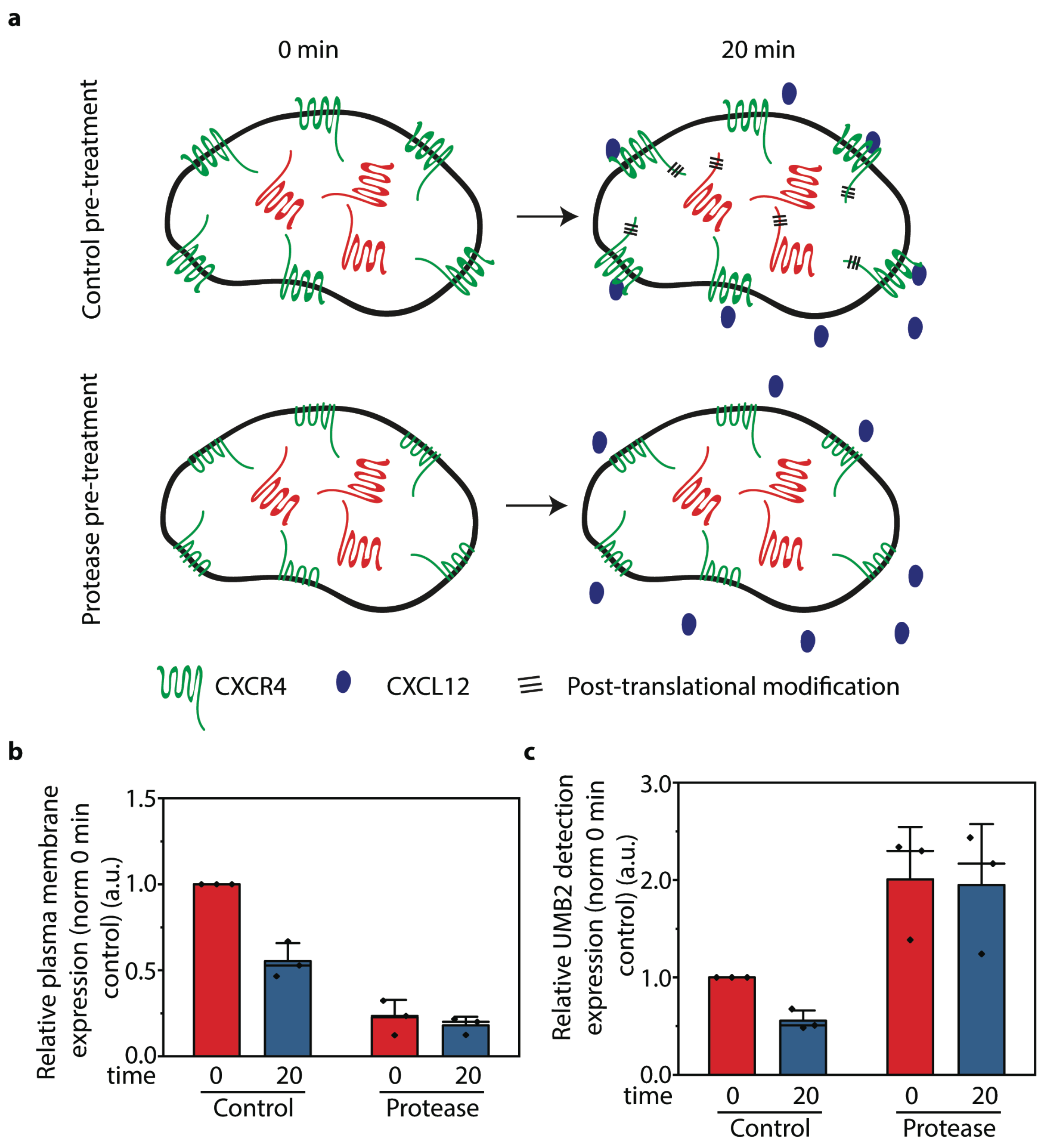

Supplementary Fig. 3: Functional plasma membrane proteins are required for CXCR4 PTM. **a** Experimental design schematic for the protease cleavage assay. Cells were pretreated with a protease (pronase 0.11% diluted in PBS) to remove extracellular motifs of transmembrane plasma membrane proteins. Afterwards, cells were stimulated with CXCL12 and plasma membrane CXCR4 and UMB2 detection was measured by flow cytometry. **b** Flow cytometry analysis of protease treatment on functional CXCR4 plasma membrane localization. Plasma membrane expression was measured using a FLAG antibody that detected an N-terminal FLAG-tagged CXCR4 and normalized to total

CXCR4 expression. Data was normalized to the 0 min control sample. **c** Flow cytometry analysis of protease treatment on CXCR4 UMB2 detection. Relative UMB2 detection was calculated by dividing UMB2 signal by total CXCR4 signal (FLAG antibody) and then normalized to the 0 min control sample. An increase in UMB2 detection in the protease treatment sample is expected as the detectable total CXCR4 population is reduced due to protease cleavage of the N-terminal CXCR4 FLAG tag. All experiments were conducted in RPE cells overexpressing WT CXCR4 and stimulated with 12.5 nM CXCL12 for the stated time course. Individual data points from each experiment are plotted; mean, SD, median line.

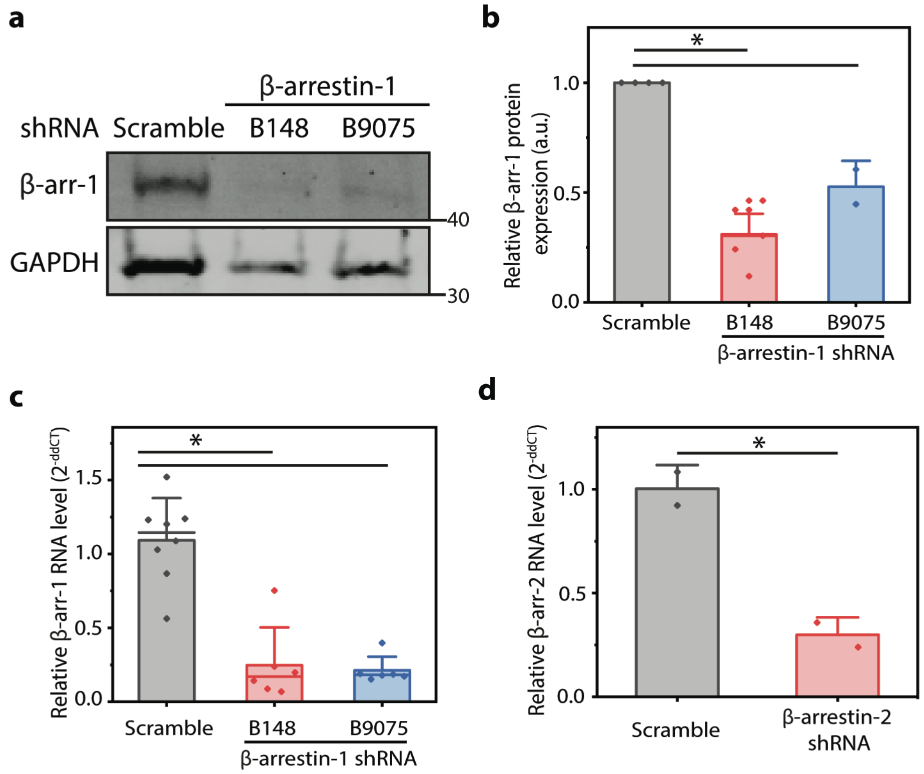

Supplementary Fig. 4: β-arrestin-1 knockdown confirmation. **a** Representative western blot illustrating relative β-arrestin-1 knockdown with two different shRNAs. **b** Quantification of β-arrestin-1 knockdown efficiency by western blot. Protein knockdown was calculated by dividing normalized β-arrestin-1 detection with β-arrestin-1 shRNA with scramble shRNA. **c-d** Relative β-arrestin-1 and β-arrestin-2 transcript levels were calculated using the ΔΔct method normalized to GAPDH and the scramble shRNA control. All experiments were conducted in RPE cells overexpressing WT CXCR4 and stimulated with 12.5 nM CXCL12 for the stated time course. Individual data points from each experiment are plotted; mean, SD, median line. Statistical significance (*) denotes $p < 0.05$. Complete raw blots are shown in Supplementary Fig. 11.

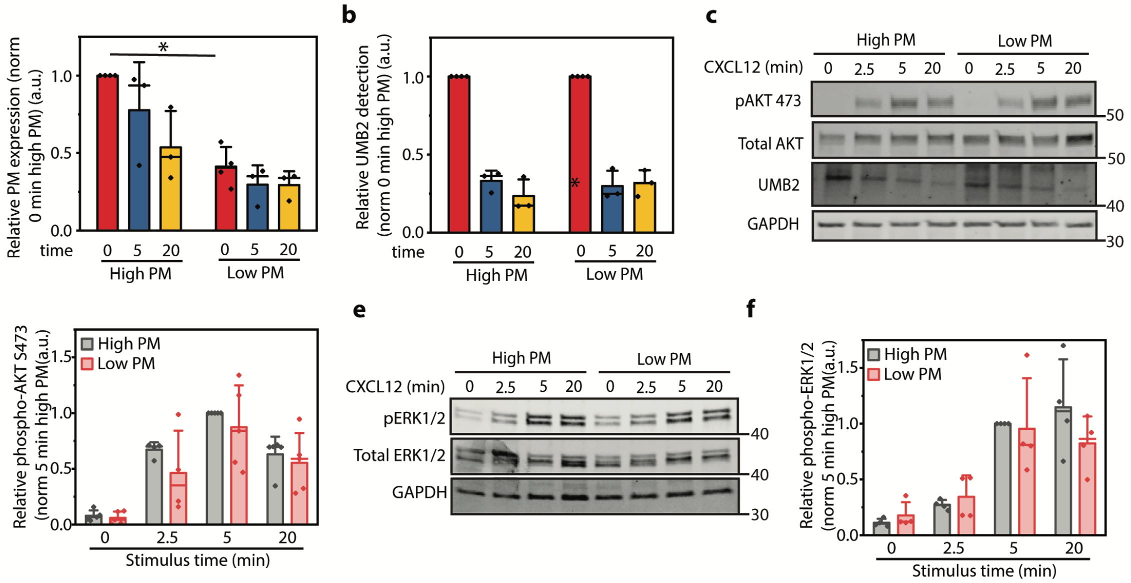

Supplementary Fig. 5: Modulation of CXCR4 plasma membrane localization does not affect CXCL12-dependent AKT S473 and ERK1/2 phosphorylation or CXCR4 PTM. **a** Flow cytometry analysis of CXCR4 plasma membrane expression of high and low plasma membrane (PM) expressing CXCR4 constructs. Plasma membrane expression was normalized to total CXCR4 expression and secondly to the 0 min high plasma membrane CXCR4 sample. **b** Flow cytometry analysis of high and low plasma membrane expressing WT CXCR4 constructs. UMB2 detection was normalized to total CXCR4 expression and secondly to the 0 min control samples. **c** Representative western blot illustrating CXCL12-induced AKT S473 phosphorylation and UMB2 detection in high and low plasma membrane expressing CXCR4 cells. **d** Western blot quantification of agonist-induced AKT S473 phosphorylation. Data was normalized to phospho-AKT S473:total AKT and to 5 min high plasma membrane expression sample. **e** Representative western blot illustrating CXCL12-induced ERK1/2 phosphorylation in high and low plasma membrane expressing CXCR4 cells. **f** Western blot quantification of CXCL12-induced ERK1/2 phosphorylation. Data was normalized to phospho-ERK/12:total ERK1/2 and to 5 min high plasma membrane expression sample. All experiments were conducted in RPE cells overexpressing WT CXCR4 and stimulated with 12.5 nM CXCL12 for the stated time course. Individual data points from each

experiment are plotted; mean, SD, median line. Statistical significance (*) denotes $p < 0.05$. Complete raw blots are shown in Supplementary Fig. 11.

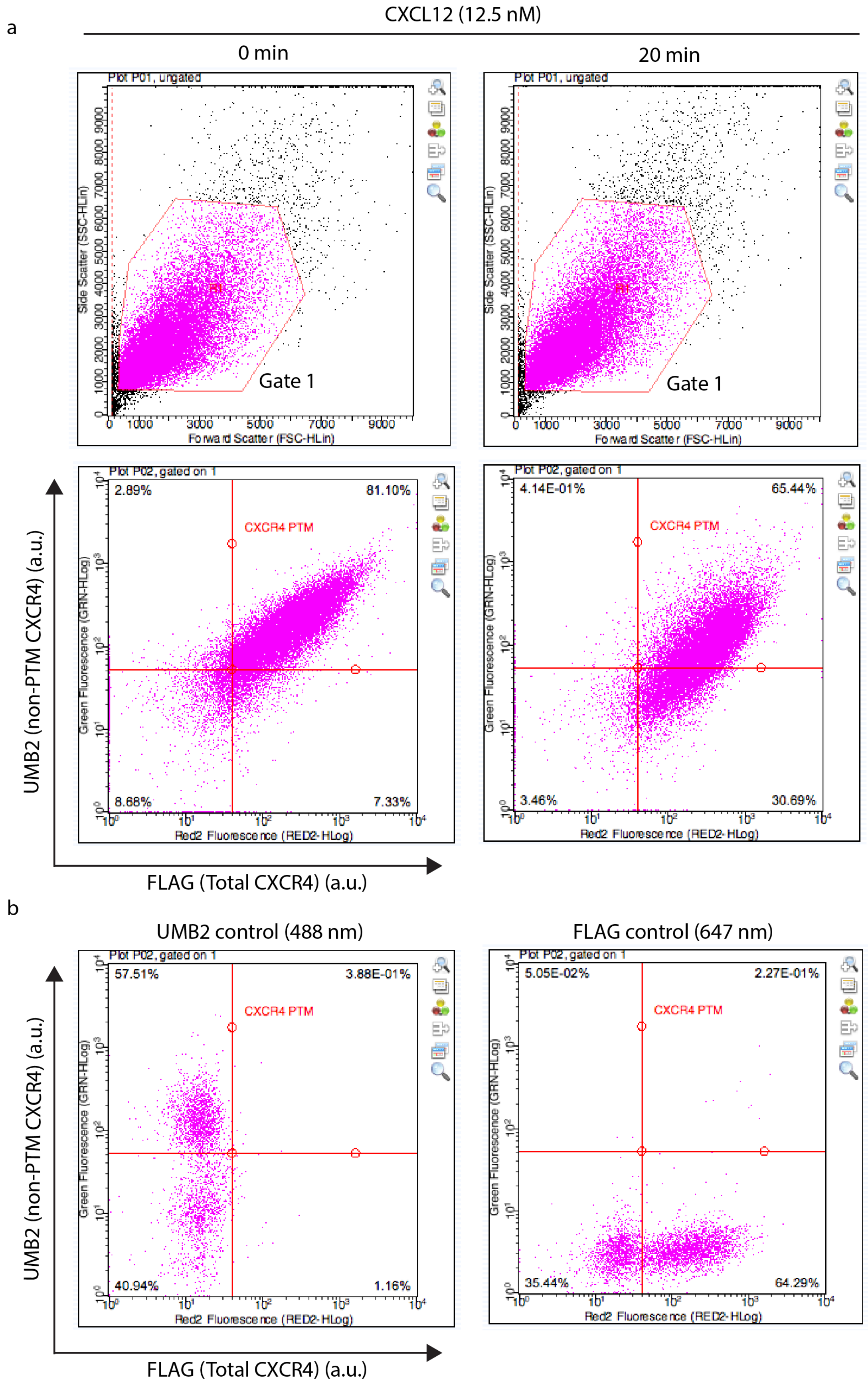
a
CXCL12 (12.5 nM)
0 min
20 min
Plot P01, ungated
Side Scatter (SSC-HLin)
Forward Scatter (FSC-HLin)
Gate 1
Plot P02, gated on 1
2.89%
81.10%
8.68%
7.33%
4.14E-01%
65.44%
3.46%
30.69%
CXCR4 PTM
Green Fluorescence (GRN-HLog)
Red2 Fluorescence (RED2-HLog)
UMB2 (non-PTM CXCR4) (a.u.)
FLAG (Total CXCR4) (a.u.)
b
UMB2 control (488 nm)
FLAG control (647 nm)
57.51%
3.88E-01%
40.94%
1.16%
5.05E-02%
2.27E-01%
35.44%
64.29%

Supplementary Fig 6: Representative flow cytometry data and gating strategy. **a** Representative SSC and FSC for flow cytometry analysis. Gate 1 is defined by the red polygon. Two samples are shown from the same experiment investigating UMB2 detection. The second row of plots correspond to the UMB2 and FLAG detection of CXCR4 in RPE cells overexpressing CXCR4 during the CXCL12 stimulus time course. Quadrants are shown to illustrate that while total CXCR4 detection remains constant, UMB2 detection decreases upon CXCL12 stimulation. X and Y axis are labeled accordingly. **b** Control experiments used for fluorophore compensation and deciding background fluorescence for quantification. With the exception of compensation controls (b) where only 5000 events were recorded, 25,000 events were collected in all experiments. All analyses were repeated for a minimum of 3 independent biological replicates and is stated in figure legends.

Fig. 1 Blots

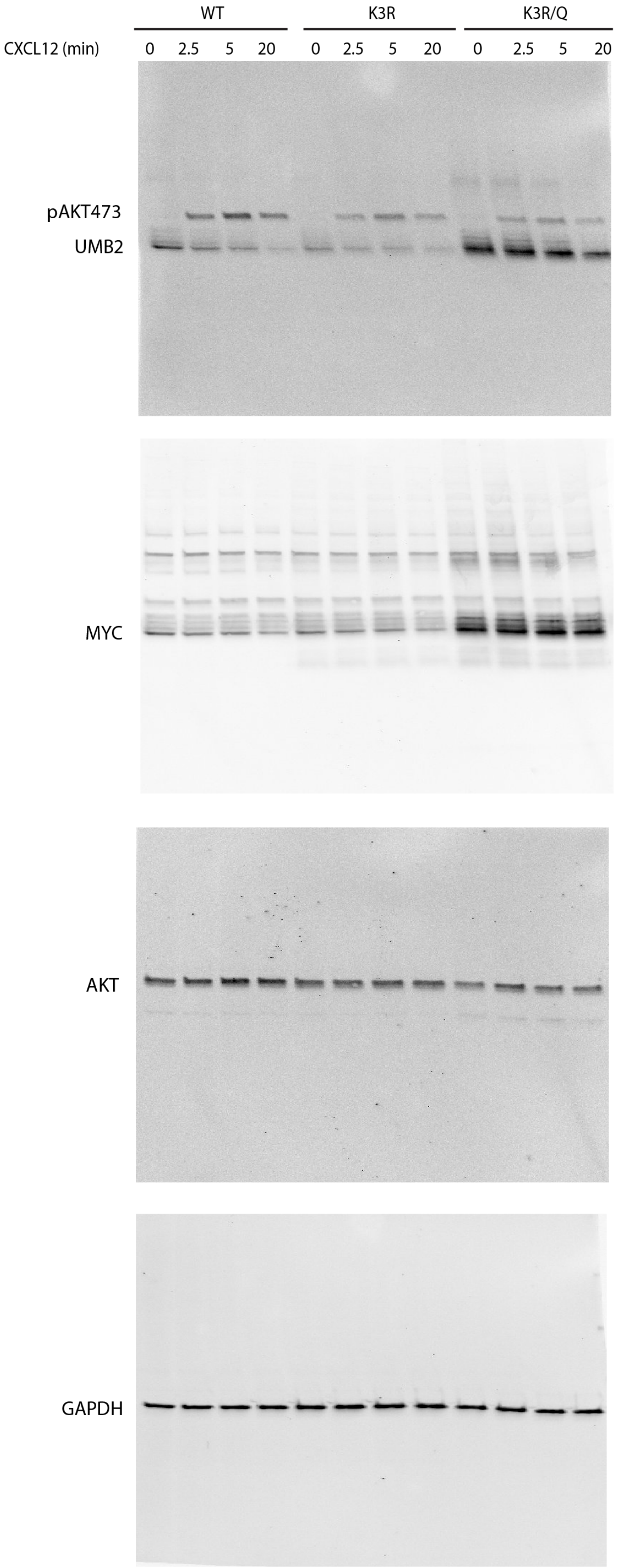
WT
K3R
K3R/Q
CXCL12 (min)
0
2.5
5
20
0
2.5
5
20
0
2.5
5
20
pAKT473
UMB2
MYC
AKT
GAPDH

Supplementary Fig 7: Complete western blots illustrated in Fig. 1. All lanes and antibodies used for staining are shown.

Fig. 2c Blots

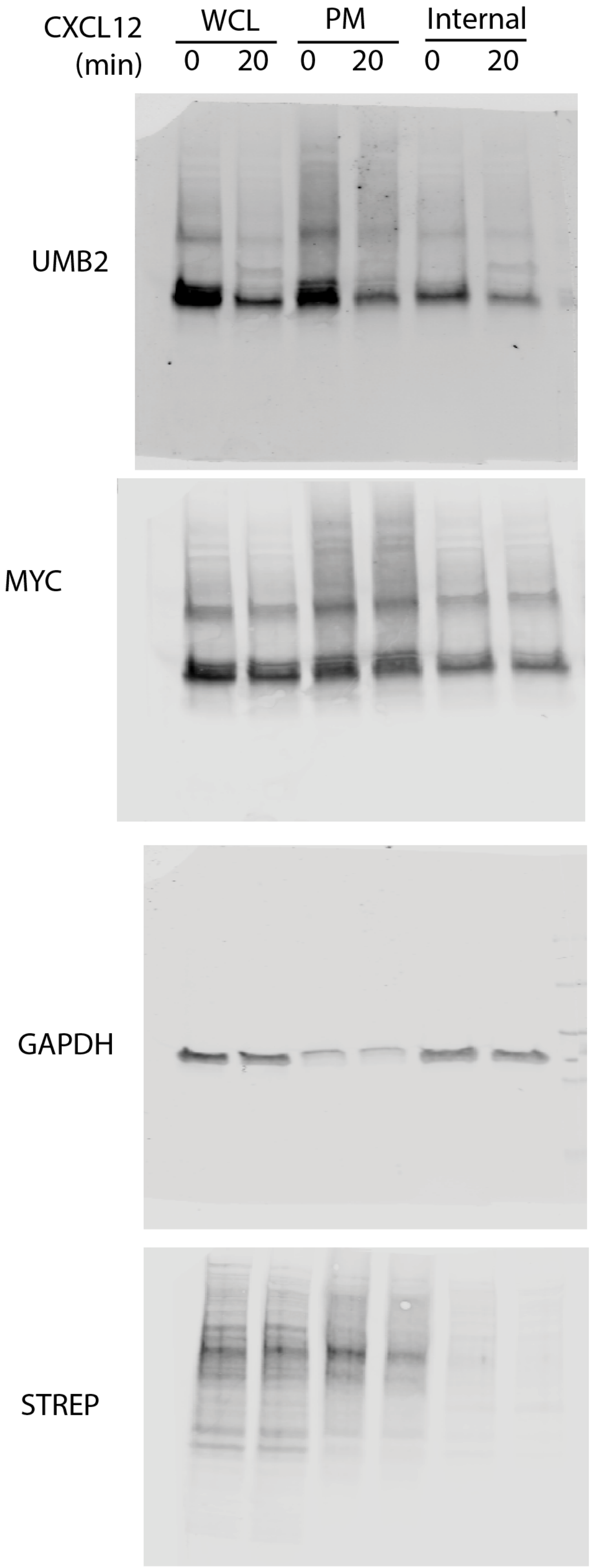

Supplementary Fig 8: Complete western blots illustrated in Fig. 2. All lanes and antibodies used for staining are shown.

Fig. 3b Blots

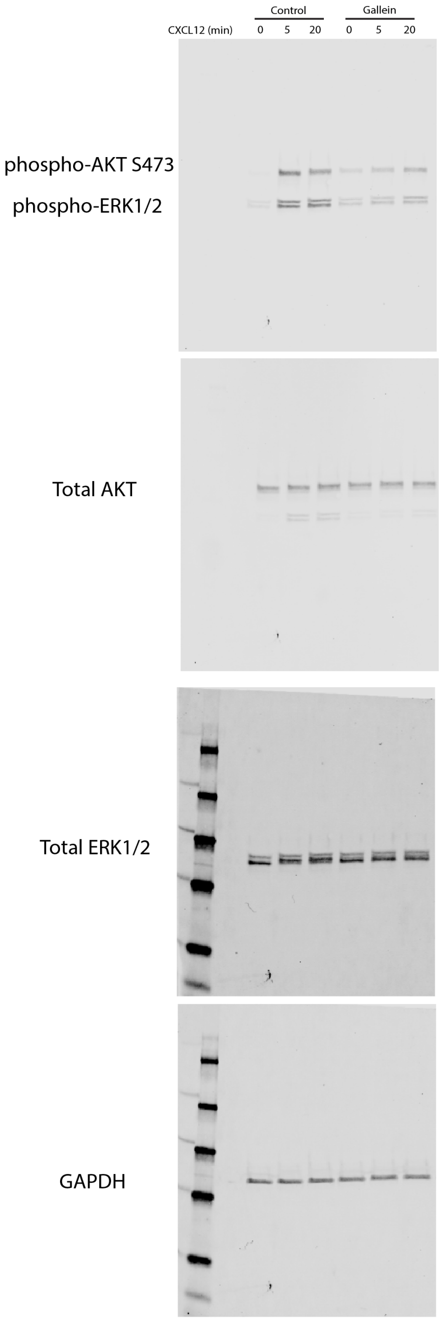

Fig. 3e Blots

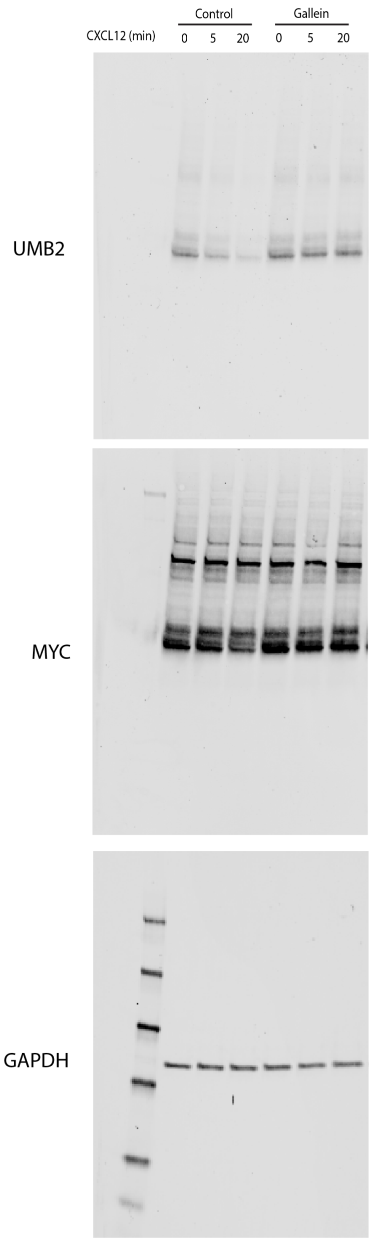

Supplementary Fig 9: Complete western blots illustrated in Fig. 3. All lanes and antibodies used for staining are shown.

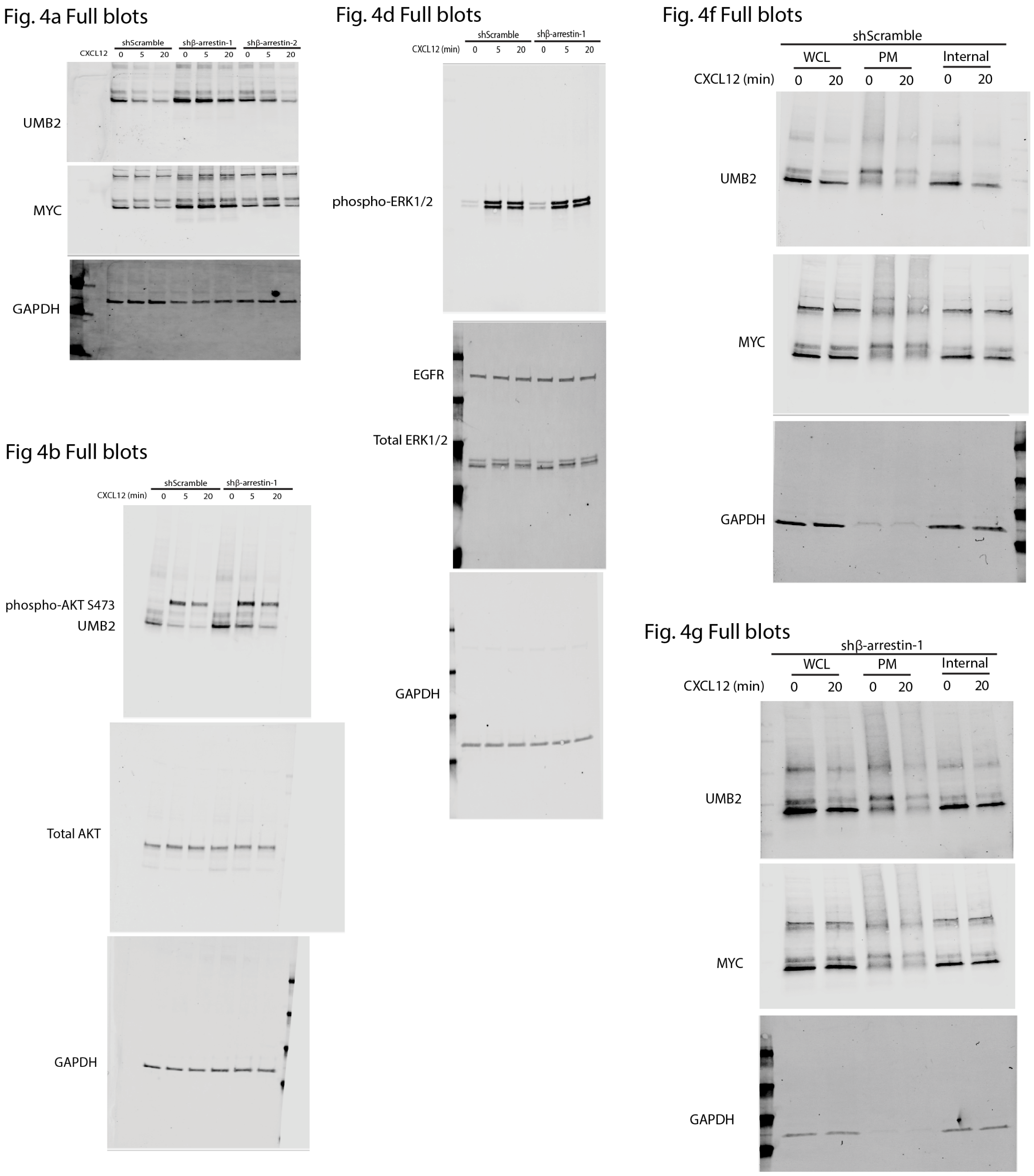

Supplementary Fig 10: Complete western blots illustrated in Fig. 4. All lanes and antibodies used for staining are shown.

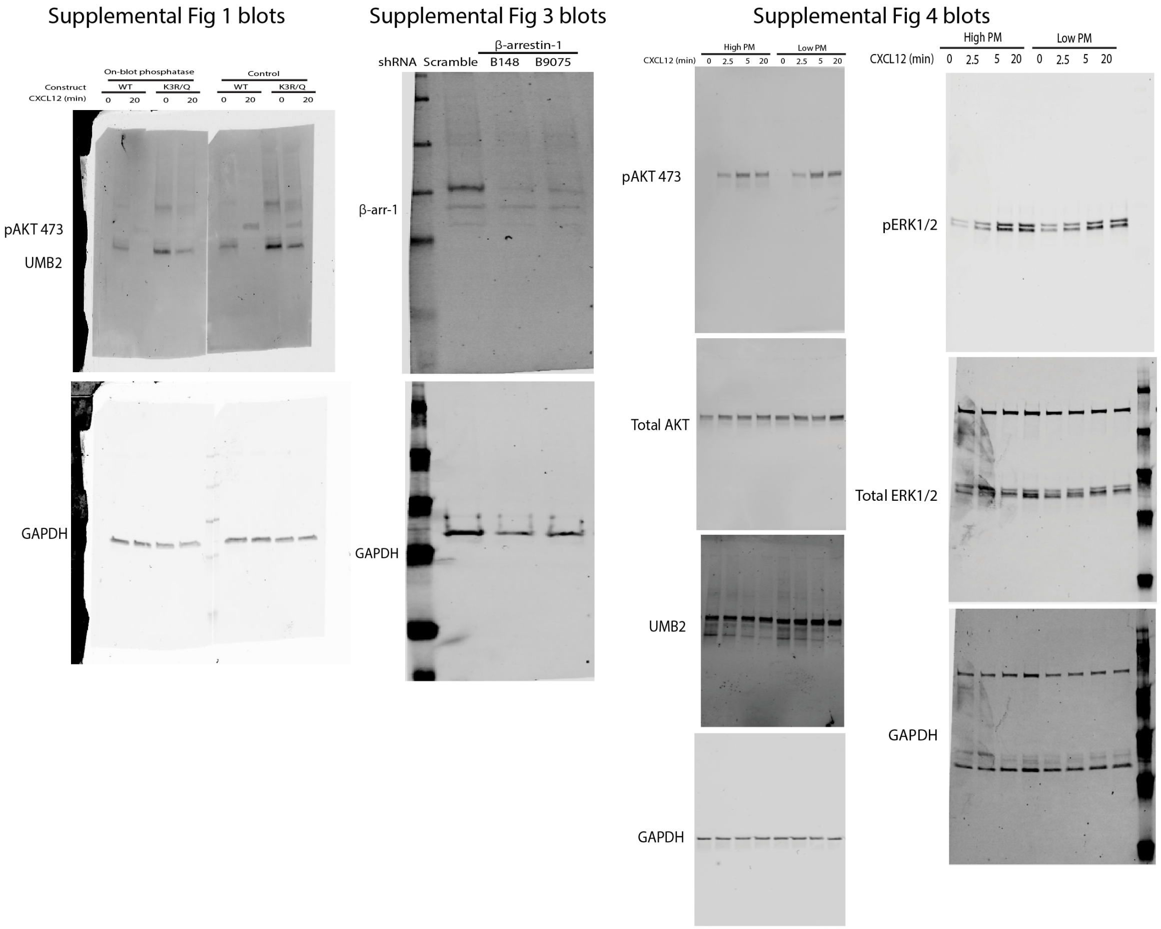

Supplementary Fig 11: Complete western blots illustrated in Supplementary Fig. 1, 3, and 4. All lanes and antibodies used for staining are shown.
